# Genetic and Physiological Responses to Heat Stress in *Brassica napus*

**DOI:** 10.3389/fpls.2022.832147

**Published:** 2022-04-05

**Authors:** Mariam Kourani, Fady Mohareb, Faisal I. Rezwan, Maria Anastasiadi, John P. Hammond

**Affiliations:** ^1^Bioinformatics Group, Cranfield University, Cranfield, United Kingdom; ^2^School of Agriculture, Policy and Development, University of Reading, Reading, United Kingdom

**Keywords:** *Brassica napus*, heat stress, flowering, alternative splicing, epigenetic modifications

## Abstract

Given the current rise in global temperatures, heat stress has become a major abiotic challenge affecting the growth and development of various crops and reducing their productivity. *Brassica napus*, the second largest source of vegetable oil worldwide, experiences a drastic reduction in seed yield and quality in response to heat. This review outlines the latest research that explores the genetic and physiological impact of heat stress on different developmental stages of *B. napus* with a special attention to the reproductive stages of floral progression, organogenesis, and post flowering. Several studies have shown that extreme temperature fluctuations during these crucial periods have detrimental effects on the plant and often leading to impaired growth and reduced seed production. The underlying mechanisms of heat stress adaptations and associated key regulatory genes are discussed. Furthermore, an overview and the implications of the polyploidy nature of *B. napus* and the regulatory role of alternative splicing in forming a priming-induced heat-stress memory are presented. New insights into the dynamics of epigenetic modifications during heat stress are discussed. Interestingly, while such studies are scarce in *B. napus*, opposite trends in expression of key genetic and epigenetic components have been identified in different species and in cultivars within the same species under various abiotic stresses, suggesting a complex role of these genes and their regulation in heat stress tolerance mechanisms. Additionally, omics-based studies are discussed with emphasis on the transcriptome, proteome and metabolome of *B. napus*, to gain a systems level understanding of how heat stress alters its yield and quality traits. The combination of omics approaches has revealed crucial interactions and regulatory networks taking part in the complex machinery of heat stress tolerance. We identify key knowledge gaps regarding the impact of heat stress on *B. napus* during its yield determining reproductive stages, where in-depth analysis of this subject is still needed. A deeper knowledge of heat stress response components and mechanisms in tissue specific models would serve as a stepping-stone to gaining insights into the regulation of thermotolerance that takes place in this important crop species and support future breeding of heat tolerant crops.

## Introduction

### Our Changing Climate and the Potential Impacts on Crop Productivity

Over the last century, global warming has become a major environmental challenge triggered anthropogenic emissions of greenhouse gases driving the continuous rise in ambient temperature (Pachauri et al., 2014; Zandalinas et al., 2021). Furthermore, the climate projections for 2100 suggest an increase in the number of drought affected areas by 50% (Battisti and Naylor, 2009). The combination of these environmental changes has the potential to destabilize plant growth and development and could result in drastic yield reductions (Lamaoui et al., 2018). Although the impact of elevated CO_2_ on crops is still under debate with potential benefits on photosynthesis, Frenck et al. (2011) showed that rising CO_2_ could not compensate for the negative impact on yield caused by high temperature stress.

On a global scale, environmental climate change is aggravating different abiotic stresses, including heat and water deficit across many biomes. Plants, being sessile organisms, cannot escape their environment and must endure a wide variety of stresses (Saraswat et al., 2017). Although plant stress responses are dynamic and encompass a complex cross-talk between different molecular and cellular interactions to establish physiological and morphological adaptations (Krasensky and Jonak, 2012), temperature exceeding the threshold of adaptation will substantially influence plant survivability (Hasanuzzaman et al., 2014). In particular, this affects cellular functioning, which in turn, hinders plant growth and development and weakens resilience to biotic stresses (Ahmed et al., 2020).

Several studies have demonstrated the effect of heat stress, often in combination with drought or other stresses, on crop production and its impact on plant metabolism, reproduction, and physiology. These studies have reported an extensive reduction in yield of various crops (Frenck et al., 2011; Hasanuzzaman et al., 2014). Lesk et al. (2016) studied the influence of extreme weather disasters on global crop production and found that, between 1964 and 2007, 1.82 and 1.19 billion metric tons of cereals were lost due to droughts and extreme heat respectively. Using four different process-based crop models, Zhao et al. (2017) demonstrated that for each 1°C rise in global mean temperature would cause a reduction in the annual production of wheat, rice, maize and soybean. In addition to its impact on yield, the increase in climate temperature has also been found to negatively impact the quality of oil in oilseed crops such as soybean, oilseed rape and sunflower, where a negative correlation between temperature and the proportion of essential fatty acids such as C18:2 and C18:3 was reported (Werteker et al., 2010; Namazkar et al., 2016). Thus, the effect of climate change is inevitably impeding vital components of human diet and nutrition.

Oilseed rape (*Brassica napus* L.) is the second-largest source of vegetable oil after soybean worldwide (United States Department of Agriculture [USDA], 2021). Given its eco-physiological adaptations to temperate climates, it is currently considered the predominant oil crop in Europe (Namazkar et al., 2016; Sun et al., 2017). *B. napus* belongs to the genus *Brassica* from the mustard Family Brassicaceae (Liu G. et al., 2018), where selection has given rise to several agronomically important lines such as oilseed rape, rutabaga, fodder rape, and kale morphotypes (Chalhoub et al., 2014). These are cultivated globally for vegetables, good quality oil, fodder, and bio-diesel industries (Chalhoub et al., 2014; Pokharel et al., 2020). As a cool-season crop, Brassica crops can be extremely sensitive to increasing temperatures, which significantly affect its production (Yu et al., 2014; Ahmed et al., 2020). Though studies in different Brassica species have found negative relationships between heat stress and seed yield and quality, the underlying mechanisms are still not well understood and require further investigation (Yu et al., 2014). Thus, to increase resilience against adverse environmental growth conditions and to maintain a stable oil supply to the global population, understanding and improving heat tolerance in *B. napus* is critical.

The acclimation mechanisms of plants to heat stress are complex and mediated by the activation of different physiological, cellular, and metabolic processes depending on their developmental stage (Zandalinas et al., 2018). These mechanisms normally encompass a combination of stress avoidance and tolerance (thermotolerance) approaches that vary with genotypes (Chaves et al., 2002). At a molecular level, the abundance in transcriptomic data facilitates the analysis of the expression of genes that play regulatory roles in different stages and tissues of plant growth and development under various stress conditions. However, the challenge lies in linking this information with the relevant biological processes taking place during stress (Chaves et al., 2009). So, uncovering the controlling mechanisms for heat tolerance is therefore imperative for successful breeding programs and future production sustainability.

In this review, we outline the latest research exploring heat stress responses in *B. napus*, focusing on the physiological impact at different developmental stages along with their underlying mechanisms. We further explore new insights into the dynamics of epigenetic changes and systems biology approaches of this plant species under heat stress. However, due to the scarcity of information specific to *B. napus*, many of the reviewed findings stem from research on the model organism *Arabidopsis thaliana* and other Brassica species.

## Physiological Impact of Heat Stress at Different Developmental Stages

### Impact of Heat Stress on Seedling and Vegetative Growth

**FIGURE 1:**
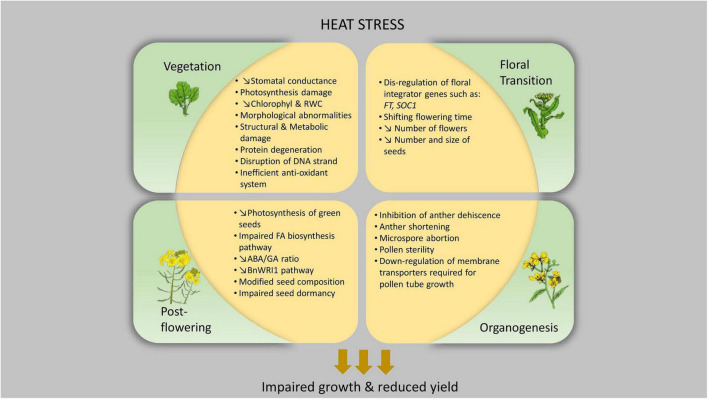
Physiological impact of heat stress at different developmental stages of *Brassica napus.*

As thermotolerance is regulated both developmentally and tissue-specifically, addressing the impact of high temperature on different developmental stages is imperative to produce heat-tolerant cultivars capable of coping with heat stress (Figure 1). Different optimum temperatures also differ considerably among species or genotypes (Angadi et al., 2000; Baux et al., 2013). In general, heat stress takes place when soil and ambient temperature is beyond a plants’ growth and photosynthetic temperature range such that, permanent harm occurs. A plants performance under heat stress is also influenced by the concomitant presence of other types of stress, such as drought (Frenck et al., 2011).

Under heatwave conditions during early vegetative growth, well-watered *B. napus* experiences an increase in saturating light (*A*_*sat*_), transpiration (*E*) and in the ratio of intercellular to ambient CO_2_ concentration (*C_*i*_/C_*a*_*) (Dikšaitytė et al., 2019). The increase in ambient temperature enhances photosynthesis performance and increases above-ground growth. However, in the presence of drought, the combined stress conditions lead to severely impaired photosynthesis, which is accompanied by a profound decrease in stomatal conductance (Dikšaitytė et al., 2019). To avoid overheating, plants open their stomata and increase transpiration, thus preventing leaf damage. In contrast, drought triggers stomatal closure as a conservative mechanism to protect plants from excessive water loss (Elferjani and Soolanayakanahally, 2018). During the flowering stage, *B. napus* responds to combined heat and drought stress by a rapid closure of their stomata. This avoids further loss of water through transpiration at the expense of evaporative cooling. Consequently, this results in an excessive increase in leaf temperature and more photosynthetic damage (Elferjani and Soolanayakanahally, 2018). This s paradox requires plants to maintain a balance between avoiding overheating and minimizing water loss (Zandalinas et al., 2018).

Additionally, impairment of chlorophyll biosynthesis and disruption of the biochemical reactions of photosystems were exhibited in 10-day-old *B. napus* seedlings subjected to 38°C (Hasanuzzaman et al., 2014). This was manifested through a significant reduction in chlorophyll and leaf relative water content as well as through an inefficient antioxidant defense system (Hasanuzzaman et al., 2014). During heat stress, the physiological changes that occur at the level of photosynthesis and transpiration alter the synthesis and usage of assimilates, which in turn translate into morphological abnormalities in the floral organs (Echer et al., 2014).

At the molecular level, heat stress can either induce structural damage through altering membrane proteins and stability or metabolic damage through disrupting enzymatic activities and production of toxic metabolites (Mittler et al., 2012). In *A. thaliana*, heat stress induced Reactive Oxygen Species (ROS) production and subsequently a higher lipid peroxidation and membrane injury (Rocco et al., 2013). Moreover, protein degradation, pigment bleaching and disruption of DNA strands are also consequences of heat stress (Mathur and Jajoo, 2014). Therefore, heat stress during vegetative and seedling growth works as an induction point for a cascade of events that can impact on reproductive organs later in development and consequently lead to reduced productivity.

### Impact of Heat Stress on Reproduction

Since the developmental progression of plants from seed to seed is bound to the success of sexual reproduction, susceptibility to heat stress at this stage can have deleterious implications on yield potential. Plants exhibit higher sensitivity to extreme temperature fluctuations during the reproductive stage of development than during their vegetative growth, often leading to impaired growth and reduced seed production (Zhang et al., 2017; Lamaoui et al., 2018), especially during meiosis and fertilization (Zhang et al., 2017), with the male reproductive organs being the most vulnerable (Giorno et al., 2013). This sensitivity fluctuates within different reproductive processes, such as: pollen development, pollen tube growth, pollen/pistil interactions, fertilization and embryo development (Erickson and Markhart, 2002; Young et al., 2004; Baron et al., 2012; Giorno et al., 2013; Zhang et al., 2017). The after-effects of heat stress during reproduction influence plant physiology in three main areas: reduction in the number of flowers at pre-anthesis, reduction in flower fertility, and poor pods and seeds development (Morrison and Stewart, 2002). Nevertheless, this has been poorly studied and specific responses at each of these processes remain elusive. Similarly, in *A. thaliana* only a limited number of reports addressed the effect of heat stress on its reproductive development (Antoun and Ouellet, 2013; Guan et al., 2014; Bac-Molenaar et al., 2015; Chao et al., 2017). Under short-term heat exposure, a genome-wide association (GWA) mapping approach in *A. thaliana* revealed different QTLs associated with heat response at pre- and post- anthesis, suggesting that these genetic regulators are developmental stage-specific (Bac-Molenaar et al., 2015). Moreover, when *A. thaliana* was exposed to 37°C for 24 h, it exhibited a severe growth impairment of stamen filaments which prevented pollination (Baron et al., 2012). At the structural level, heat stress caused abnormalities in the reproductive organs including vacuolation, collapse within microspores and abnormally shaped pollen sacs, incapable of accumulating carbohydrates (Baron et al., 2012). In spring wheat, pre-anthesis associated events such as gametogenesis and gamete development appeared highly sensitive to high-temperature treatment as heat stress resulted in reduced floret fertility and consequently a significant reduction in seed number and weight (Bheemanahalli et al., 2019).

#### Floral Progression

**FIGURE 2:**
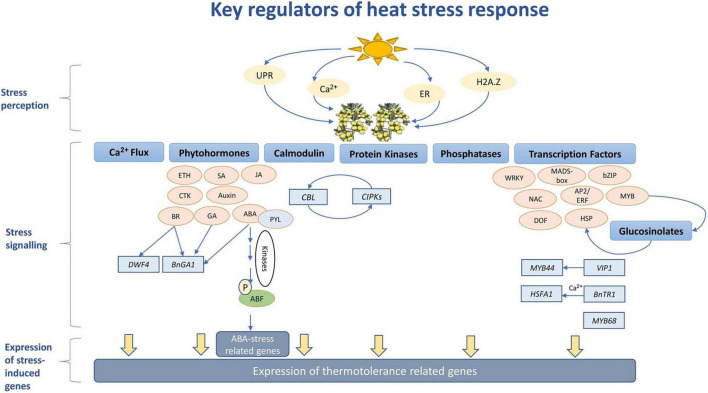
Major flowering time regulators in *Brassica napus.*

In agriculture, flowering is a key prerequisite stage for the transition from the vegetative phase to reproduction. It is regulated by the expression of a set of floral integrator genes such as *FLOWERING LOCUS T (FT)*, to activate the meristem identity genes *LEAFY (LFY), APETALA1 (AP1), SEPALLATA3 (SEP3)* and *FRUITFULL (FUL)*, causing the transition from vegetative to a floral meristem (Blümel et al., 2015). Under different stress conditions, plants utilize different mechanisms to alter flowering time (FTi) as a strategy to ensure the success of reproduction (Figure 2; Simpson, 2004; Ko et al., 2010; Blümel et al., 2015; Kazan and Lyons, 2016). However, these mechanisms are known to impact crop productivity (Kazan and Lyons, 2016).

During flowering, energy demand spikes due to different processes, such as: formation of flowers, nitrogen use efficiency and reduced photosynthesis (Schiessl, 2020). Such crucial physiological period has been found sensitive to various biotic and abiotic stress and particularly to heat, which has a pronounced effect on the timing of flowering, depending on its duration, frequency, and intensity (Lohani et al., 2020b). In a split-plot experiment, different genotypes of *B. napus* were exposed to high-temperature stress (31°C/14°C - day/night) during flowering across two sowing periods (Koscielny et al., 2018). Results showed a reduction in yield by 55% in the heat treatment during winter and by 41% during the subsequent autumn experimental run (Koscielny et al., 2018). At different developmental stages, severe damage to reproductive organs of three Brassica species (*B. juncea* L., *B. napus* L., *B. rapa* L.) occurred when exposed to 35°C/15°C for 7 days resulting in yield reductions (Angadi et al., 2000). It was noted that yield was affected by heat stress at flowering more than at pod development, indicating a vital developmental threshold passed by pods rendering them more heat tolerant. Interestingly, among the three studied Brassica species, only *B. napus* could not recover completely after heat stress was removed, producing abnormal, plump, and short pods (Angadi et al., 2000). A reduction in seed yield was observed following 29.5°C temperature treatment during flowering, primarily due to the reduction in the number of flowers and in the number and size of seeds produced per flower (Morrison and Stewart, 2002).

During flower opening in rice air temperature is crucial in determining the fate of reproduction (Bheemanahalli et al., 2017) and the resulting earlier or delayed flowering can negatively impact crop growth and quality. In a recent study carried out by Pokharel et al. (2020), high night temperature stress-induced a shift in *B. napus* peak flower opening time into earlier and cooler morning hours, suggesting an adaptation toward the heat escape response, yet this was accompanied by a significant reduction in yield. Whilst *B. napus* is not strictly dependent on pollinators, it has been shown that pollinators enhance seed yield and quality (Zou et al., 2017). So, shifting flowering time drives the flower opening out of sync with pollinator activity, and could potentially lead to reductions in yield.

Although FTi regulator genes and their associated networks have been well characterized in *A. thaliana*, the conservation of these genes in other Brassica species indicates that their regulation takes place in the same way as in this model system. However, environmental adaptation has led to the evolution of new FTi regulator genes with no functional equivalence in *A. thaliana* (Blümel et al., 2015). Moreover, some FTi gene copy numbers were found to either change or lose functionality over evolutionary periods resulting in sub-functionalization (Schiessl, 2020). As a result, it is difficult to functionally characterize crop FTi regulators and pathways based on inferences from this model species. This was manifested in a study by Del Olmo et al. (2019) when *B. rapa* delayed flowering was associated with reduced *BraA.FT.a* mRNA levels upon high-temperature treatment. High levels of H2A.Z were also found to occupy the *BraA.FT.a* locus, which in turn affected chromatin conformation and hindered its accessibility. The same conserved chromatin regulatory mechanism in *A. thaliana* showed an opposite trend and resulted in accelerated flowering (Del Olmo et al., 2019). This brings attention to the fact that thermosensory pathways perform differently in different crops to alter FTi regulators.

Considering the scarcity of studies addressing the impact of increased ambient temperature on FTi-related signaling pathways in *B. napus*, the continuous rise in global temperature will need to drive future research to explore these pathways and the underlying regulators under heat stress. Such studies will help identify key regulators, direct genetic modifications to candidate genes involved in these pathways and to provide more reliable data for breeding programmes to deliver heat stress tolerant cultivars.

#### Organogenesis

The next stage after floral transition is the differentiation of floral organs or organogenesis. In *B. napus* as in other flowering plants, gametogenesis and reproduction are highly vulnerable to high-temperature stress (Angadi et al., 2000; Young et al., 2004). From inhibition of anther dehiscence and anther shortening to reducing pollen germinability and viability (Rahaman et al., 2018), the impact of heat stress on different reproductive organs has been reported in various species, such as: wheat, soybean, Brassicas and tomato (Giorno et al., 2013; Draeger and Moore, 2017; Djanaguiraman et al., 2019). Moreover, heat stress during microsporogenesis leads either to microspore abortion or pollen sterility (Lohani et al., 2020b). The variety of affected species suggests the existence of common processes involved in heat stress-related infertility (Kim et al., 2001; Rang et al., 2011; Sage et al., 2015) and yield loss (Morrison and Stewart, 2002; Frenck et al., 2011; Zhao et al., 2017). However, in *B. napus*, such processes are still not very well studied.

In *Arabidopsis thaliana*, during anther development, heat stress causes male sterility in a stage-specific manner (Kim et al., 2001). Disruption of male meiotic processes was associated with abnormal pollen development and morphological analysis with a failure in the separation of pollen mother cells and a subsequent microspore differentiation (Kim et al., 2001). Heat stress also caused a dramatic down-regulation in membrane transporters required for pollen tube growth such as K^+^ and carbohydrate co-transporters, which would adversely impact pollination (Poidevin et al., 2020). To discover the effect of heat stress after flowering initiation in *B. napus*, Young et al. (2004) reported that following heat exposure at 35°C, both micro and megagametophytes were found thermosensitive and resulted in a significant reduction in pollen viability and germinability as well as in seed development. An increase in temperature was also reported to induce sterility and lead to a decrease in fertility rate, pod-setting ratio and seed number per pod (Zhang et al., 2012). Similarly, in a comparative transcriptomic analysis, Tang et al. (2019) found that genes encoding heat shock proteins, skeleton proteins, GTPase and calmodulin were potentially involved in the mechanism of thermosensitive genic male sterility (TGMS) under high temperature inducement. Moreover, genes encoding plant hormone signaling pathways (auxin, gibberellins, jasmonic acid, abscisic acid, brassinosteroid signalings), along with some well-known transcription factors (MADS, NFY, HSF, MYB/C, and WRKY) were also found involved in the regulation of TGMS in the flowers grown under 25°C (Tang et al., 2019).

Although many studies have reported that male reproductive organs are more sensitive to heat stress than female reproductive organs, changes in the female reproductive organs following heat stress have also been documented (Hedhly, 2011). Additionally, according to the identity of the cell, the female gametophyte possesses a unique and differentially mediated response to heat stress (Ambastha and Leshem, 2020). These findings suggest that temperature plays a vital role in affecting the fertility of pollen and the morphology of the flower reproductive organs.

#### Post-flowering

The thermotolerance of seed setting rate and filling, are also crucial in determining grain yield and composition. During later reproductive growth, the biosynthesis and deposition of oils and proteins in seeds take place at the seed filling stage. Hence, it is imperative to strictly regulate this stage for the generation of high-quality and balanced oil (Brunel-Muguet et al., 2015).

Photosynthesis in *B. napus* green seeds and pods induces the formation of the necessary components of the fatty acid biosynthesis pathway (Huang et al., 2019). This pathway was found to be regulated by the transcription factor BnWRI1 (Wu et al., 2014). In a recent study, heat stress suppressed oil deposition in developing seeds and impaired fatty acid biosynthesis where the inhibition of the *de novo* fatty acid biosynthesis pathway was associated with the inhibition of photosynthesis and downregulation of many genes in the BnWRI1 pathway (Huang et al., 2019). These findings have highlighted the crucial function of BnWRI1 as a key regulator within the intricate regulatory systems of heat stress and biosynthesis of storage compounds. Brunel-Muguet et al. (2015) also showed that high temperature during seed development modified seed composition and impaired the acquisition of seed dormancy. This was accompanied by a decrease in abscisic acid/gibberellic acid (ABA/GA) ratio, which plays a role in controlling seed composition and storage capacity (Brunel-Muguet et al., 2015). Similarly, the total lipids and all the fatty acids studied were significantly lower in *B. napus* seed oil when temperature was raised from 19°C to 24°C, resulting in imbalanced proportions of essential fatty acids (Namazkar et al., 2016).

Interestingly, not only high day temperature was found to impact seed quality and yield, but also high night temperature was reported to have detrimental effect on yield and seed fatty acid composition in *B. napus*. During flowering and silique-filling stages, Pokharel et al. (2020) reported that high night temperature caused significant alterations in the reproductive organs and led to a substantial reduction in seed yield and number of siliques, which was further explained by the thermal impairment of photosynthetic assimilation enzymes. Likewise, exposure of susceptible *B. napus* cultivars to high night temperature stress between flowering and seed filling stages also induced a profound reduction in total fatty acids and in fatty acid composition in seeds (Zhou et al., 2018). It is worth noting that the magnitude of heat stress and the cardinal temperature unequivocally contribute to the degree of impact on each crop species (Mittler et al., 2012).

In summary, high-temperature stress induces adverse effects on different processes and organs during *B. napus* reproduction. From floral transition to seed filling, such environmental threats will eventually translate into unfavorable agronomic and quality traits and ultimately challenge the production of seed oil with strong nutritional values. Knowledge about heat stress during these stages is limited and more insight on this topic will help scientists and breeders to design strategies to protect this important crop species from the impact of the stress potentially caused by climate change presently and in the future.

### Impact of Heat Stress on Postharvest Seed Storage

During postharvest storage, high-temperature stress has also been found to impact the safety and nutritional qualities of stored seeds (Krasucki et al., 2002). Brassica seeds are rich in bioactive compounds that exhibit antioxidant action and can play a vital role in reducing blood cholesterol levels (Ratnayake and Daun, 2004). In *B. napus* seeds, the concentrations of phytosterols can be twice that of sunflower and soybean oils (Vlahakis and Hazebroek, 2000), and the content of phenolic compounds can be 10-fold greater than in seeds of other crops (Siger et al., 2018). The abundance of such important biologically active compounds is sensitive to storage conditions. High temperature and moisture content were associated with the deterioration of seeds and degradation of its biochemical contents (Gawrysiak-Witulska et al., 2012). After 18 days of storage at 25°C and 30°C, the amount of phytosterols decreased significantly, reaching 24 and 58% less in *B. napus* seeds with a moisture content of 15.5% (Gawrysiak-Witulska et al., 2012). Under similar storage conditions, tocopherols dropped by 14.4% while plastochromanol-8 loss ranged from 4 to 24% (Gawrysiak-Witulska et al., 2011). These natural antioxidants play a crucial role in determining lipid stability and inhibition of auto-oxidation in stored seeds (Olejnik et al., 1997). As a result, the degradation of such compounds not only alters the quality of oil but can also lead to the formation of oxidized derivatives with cytotoxic properties (Rudzińska et al., 2009). To investigate the effect of inappropriate storage conditions (13.5% moisture content and 25°C temperature) on seed quality in *B. napus*, Siger et al. (2018) reported an intense growth of fungi along with a decrease in sinapic acid derivatives, the main phenolic compounds in *B. napus* (Siger et al., 2018). The development of fungal growth not only reduces the time of safe storage but also causes a risk of seed deterioration and mycotoxin contamination posing a serious threat to human and animal health (Siger et al., 2018). Furthermore, fungal growth and respiration could also result in the production of additional heat and moisture in the seeds (Mills, 1989), which further exacerbates the problem of temperature stress and microbial infestation.

## Plant Response Mechanisms to Heat Stress

Plants respond to environmental adversities, such as changes in ambient temperature through a cascade of signaling pathways, resulting in cellular readjustments at the transcriptome, epigenome, proteome and metabolome levels. Eventually, these adjustments provide the required balance through multiple adaptive strategies to ensure survival under the new conditions. Such intricate machinery involves an interconnected crosstalk between different components of the cell, initiated by stress perception, carried out through stress signaling and ultimately end with the expression of a specific set of downstream stress-induced genes that result in a phenotypic response to the stress.

**FIGURE 3:**
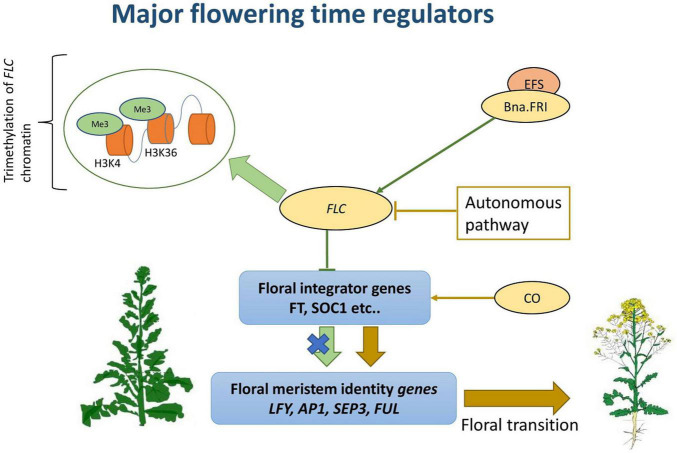
Regulatory and signaling events involved in plant heat stress response.

Different sensors are involved in the initial stress perception (Lamaoui et al., 2018; Baillo et al., 2019), and these include plasma membrane mediated calcium flux, endoplasmic reticulum (ER) and cytosolic unfolded protein response (UPR) sensors, and decreased histone occupancy in the nuclei (Mittler et al., 2012). The generated signals are then integrated through a network of multiple events that includes calcium fluxes, calmodulin, phosphatases, transcriptional regulators, hormones, and protein kinases such as CDPKs, MAPKs (Figure 3; Verma et al., 2016; Lohani et al., 2020a). Hence, the complexity of heat response lies in the broad range of pathways involved, which appear to be tissue and developmental stage specific (Zhang et al., 2017).

### Phytohormones

One of the principal regulators of abiotic stress responses are phytohormones, which encompass several compounds, such as: abscisic acid (ABA), brassinosteroids (BR), salicylic acid, jasmonates (JA), ethylene, auxins, cytokinins, and gibberellins (Verma et al., 2016). These phytohormones have essential roles in protecting plants against environmental stress (Lohani et al., 2020a). The intricate signaling role of these compounds is carried out either directly or through crosstalk between interacting arrays of various secondary messengers (Verma et al., 2016). During biotic and abiotic stress responses, ABA plays a powerful role in mediating plant adaptation to stress (Baron et al., 2012). In *A. thaliana*, temperature stress induced the differential expression of several ABA biosynthesis genes (*ABA1, ABA2, ABA4, AAO3, NCED3*) in an organ-specific manner (Baron et al., 2012; Table 1). Moreover, in *B. napus*, Di et al. (2018) identified 14 differentially expressed pyrabactin resistance 1-like (PYL) genes, which play core roles in ABA signaling networks, at different time points of heat treatment. *BnPYR1-3, BnPYL1-2, BnPYL3-1, BnPYL4-2, BnPYL5-4, BnPYL6-1, BnPYL7-2, and BnPYL 9-2* were up-regulated while *BnPYR1-4, BnPYL2-2, BnPYL4-6, BnPYL8-5, BnPYL8-6, BnPYL9-1* were down regulated (Table 1). Also, some *BnPYL* genes such as *BnPYR1-3, BnPYL1-2*, and *BnPYL7-2* had similar expression patterns under drought, salinity, and heat treatments suggesting that these genes might be important candidates for improving tolerance to abiotic stress (Di et al., 2018). In addition to its role during abiotic stress response, tissue ABA concentrations were also found to influence seed components during grain filling (Chandrasekaran et al., 2014). Under high-temperature conditions, changes in ABA content in seeds were involved in the modification of downstream expression of genes associated with the biosynthesis of seed storage compounds in castor beans (Chandrasekaran et al., 2014). Thus, the quality determining characteristics of seeds such as seed germination, vigor and storability can be negatively affected by the impact of heat stress through inducing changes in the concentration of important hormonal signaling molecules (Gao et al., 2010).

**TABLE 1:** Heat stress responsive genes identified in *Brassica napus* and related species.

Gene/gene family	Expression	Function	Species	References
*BnaDRMc*	up-regulation	DNA methylase and de-methylases	*B. napus*	Fan et al., 2020
*BnaDNMT2*	up-regulation			
*BnaDRMa*	up-regulation			
*BnaDRMg*	up-regulation			
*BnaDRMh*	up-regulation			
*BnaROS1a*	down-regulation			
*BnaCMTa*	down-regulation			
*BnaMETs*	down-regulation			
*BnaHsf15/16* (subclass A2)	down-regulation	Heat shock transcription factors	*B. napus*	Zhu et al., 2017
*BnaHsf47* (subclass B1)	down-regulation			
*BnaHsf50* (subclass B2A)	down-regulation			
*BnaHsf21/22* (subclass A4A)	up-regulation			
*BnaHsf46* (subclass B1)	up-regulation			
*BnaHsf17/18* (subclass A3)	up-regulation			
*BnaHsf43* (subclass A8)	up-regulation			
*PYR1-3*	up-regulation	ABA receptor PYR/PYL (pyrabactin resistance 1-like) family	*B. napus*	Di et al., 2018
*PYR1-4*	down-regulation			
*PYL1-2*	up-regulation			
*PYL2-2*	down-regulation			
*PYL3-1*	up-regulation			
*PYL4-2*	up-regulation			
*PYL4-6*	down-regulation			
*PYL5-4*	up-regulation			
*PYL6-1*	up-regulation			
*PYL7-2*	up-regulation			
*PYL8-5*	down-regulation			
*PYL8-6*	down-regulation			
*PYL 9-1*	down-regulation			
*PYL 9-2*	up-regulation			
*BraA.FT.a*	down-regulation	Flowering time regulator gene	*B. rapa*	Del Olmo et al., 2019
*ABA2/4* (in cauline leaves and inflorescence meristems)	up-regulation	ABA biosynthesis genes	*A. thaliana*	Baron et al., 2012
*NCED2/5/6* (in cauline leaves)	down-regulation			
*NCED5/6* (in developing siliques)	up-regulation			
*NCED3* (in cauline leaves)	down-regulation			
*NCED3* (in inflorescence meristems and developing siliques)	up-regulation			
*CYP707A1/4* (in cauline leaves)	up-regulation	ABA catabolism		
*CYP707A2/3* (in cauline leaves)	down-regulation			
*ABCG25/40* (in cauline leaves and inflorescence meristems)	down-regulation	ABA transport and homeostasis		
*UGT71B6* (in cauline leaves and inflorescence meristems)	down-regulation			
*AtBG1* (in cauline leaves)	up-regulation			
*AtBG1* (in inflorescence meristems)	down-regulation			
*BnGA1*	down-regulation	A putative G protein α subunit (Gα)	*B. napus*	Gao et al., 2010
*B. napus* thermal resistance gene 1 (*BnTR1*)	up-regulation	Membrane-bound RINGv (Really Interesting New Gene Variant) protein with E3 ligase activity	*B. napus*	Liu et al., 2014
*Br*MYB*28*	up-regulation	Transcription factors	*B. rapa*	Justen and Fritz, 2013
*Br*MYB*34*	up-regulation			
*AtHsp90*	up-regulation	Transcription factor	*A. thaliana*	Ludwig-Müller and Krishna, 2000
*BnaCBL1/9*	up-regulation	Ca^2 +^ sensors and regulators of CBL-interacting protein kinases	*B. napus*	Zhang H. et al., 2014
*BnaCBL10*	down-regulation			
*BnaCIPK3/6*	up-regulation			
*BnaCIPK1/11/12/26*	down-regulation			
*BnGLYI-3*	up-regulation	Glyoxalase System	*B. napus*	Yan et al., 2016
*BnWRI1*	up-regulation	*De novo* fatty acid biosynthesis pathway	*B. napus*	Huang et al., 2019

*Expression relates to the reported direction of transcriptional change in the reporting reference associated with heat stress.*

Although many studies addressed the role of ABA as a major stress-responsive hormone, the role of other stress hormones has also been studied in response to various abiotic stress factors. For example, high concentrations of ABA and BR and low concentrations of gibberellic acid 3 (GA3) significantly induced *BnGA1* expression, a gene that encodes a putative G protein α subunit (Gα) in *B. napus* (Gao et al., 2010). *BnGA1* was initially found strongly expressed at the bolting and fruiting stages. However, different abiotic stresses prompted different expression patterns with an apparent low expression in response to heat. This reduction in transcription could be attributed to one of the physiological events in decelerating cellular processes under temperature stress (Gao et al., 2010). BR were also found to enhance seedling tolerance to drought and cold stress and counterbalance the inhibitory impact of salt stress on seed germination in *A. thaliana* and *B. napus* (Kagale et al., 2007). Transgenic *B. napus* overexpressing the *Arabidopsis* BR biosynthetic gene *DWF4* exhibited increased seed yield and enhanced stress response to heat and drought (Sahni et al., 2016). At higher concentrations, stress hormones such as JA inhibit growth as a strategy to mobilize the metabolic reservoir between growth and stress response processes (Heinrich et al., 2013). The overexpression of BR biosynthetic gene *DWF4* induced a downregulation of several JA biosynthesis and signaling genes, suggesting a mechanism played by BR to balance the inhibitory effect of JA on growth (Sahni et al., 2016). Furthermore, analysis of global transcription profiles after heat stress showed diverse expression patterns between different hormone signaling pathways. While genes encoding proteins involved in the biosynthesis of ethylene and GAs were all reduced, genes encoding auxin synthesis, binding and transport were all induced in seeds (Yu et al., 2014). These studies suggest a complex interaction among different phytohormones, through switching the activities of certain genes as needed depending on the stress situation. Thereby, functioning as key regulators to achieve a state of homeostasis and maintain a balance between stress tolerance and plant productivity.

### Transcription Factors

Different TF families have been identified in plants such as AP2/ERF, MYB, HSP, bZIP, NAC, DOF, MADS-box, etc. (Ghorbani et al., 2020; Table 1). In a recent study, Wang et al. (2018) identified 2,167 TFs belonging to five different families in *B. napus* with 7.6% identified as novel and unique to *B. napus*. Interestingly, among the DEG identified in response to different abiotic stresses, 70% were responsive to heat after 12 h of treatment and these include: 62 BnAP2/EREBPs, 32 BnbZIPs, 61 BnMYBs, 40 BnNACs, and 27 BnWRKYs (Wang et al., 2018).

During plant heat stress, the heat shock response is a conserved response during which heat shock transcription factors (HSF) regulate heat shock proteins (HSP) through recognizing and binding to conserved palindromic motifs in the promoter region of heat-stress responsive genes (Scharf et al., 2012). As a result, HSP bind to denatured proteins and inhibit their aggregation, thus maintaining protein homeostasis and eventually thermotolerance (Su and Li, 2008). Unlike the limited number of HSF encoding genes in yeast and animals, plants possess large and diverse HSF gene families with complex species-specific functions (Scharf et al., 2012). There are 21 HSF genes in the *A. thaliana* genome (Nover et al., 2001), 25 HSF genes in the rice genome (Chauhan et al., 2011), and 56 HSF genes in the wheat genome (Xue et al., 2014). Interestingly, 64 HSF encoding genes were discovered in *B. napus*, rendering it the largest HSF family in plants identified so far (Zhu et al., 2017). The diversity of HSF genes in plants has likely arisen from whole-genome duplications events that took place during evolution (Chalhoub et al., 2014). In the case of *B. napus*, it is believed that the allopolyploidy process contributes significantly to the expansion of its HSF gene families, a key element for its adaptation and survivability under different environmental conditions (Zhu et al., 2017).

An example of a novel yet-conserved gene that plays a key role in the thermal resistance in *B. napus* is *Brassica napus* thermal resistance gene 1 (*BnTR1*). BnTR1 belongs to a new type of membrane-bound RINGv (Really Interesting New Gene Variant) protein with E3 ligase activity and was found to control the expression of HSFs related genes such as *HSFA1a* through the regulation of cytosolic Ca^2+^ concentration under heat stress, thus enhancing thermotolerance (Liu et al., 2014). It was also noticed that *BnTR1* was able to increase heat tolerance in *O. sativa* without producing any defective growth phenotypes as seen with other thermal-resistant genes, such as: *DREB1A*, *AtHSFA3* and *BhHSF1* (Yoshida et al., 2008; Hong et al., 2009; Zhu et al., 2009). Liu et al. (2014) also found that the expression of *BnTR1* increased the yield of rice suggesting its role in alleviating adverse environmental impacts on the crop through functioning as a master regulator of heat stress-responsive genes.

Other heat-responsive markers have also been identified in *B. napus* heat stress tolerance mechanisms. MYB genes were concomitantly reprogrammed and induced in the silique wall and seeds of *B. napus* following exposure to heat stress (Shamloo-Dashtpagerdi et al., 2018). After constructing a gene regulatory network of key stress regulatory genes that respond to drought and salt tolerance in *B. napus*, comparative genomic analysis from *A. thaliana* and *Eutrema salsugineum* and their *B. napus* homologs showed that transcription factor *MYB44* and its direct regulator *VIP1* had the largest number of connectivity and achieved the highest centrality values among the studied genes, implying its pivotal role in the regulatory network and ultimately in the plant stress tolerance system (Shamloo-Dashtpagerdi et al., 2018). At the physiological level, TFs also play important roles in plant growth and development. In *A. thaliana*, the ectopic expression of transcription factor *AtMYB68* in *B. napus* following severe heat stress at flowering enhanced pollen viability and led to significant improvement in yield (Deng et al., 2020). These findings show the key role of TFs in the reproductive heat tolerance system pertained by *A. thaliana* and other related species (Yu et al., 2014) and altogether suggest a conserved stress response with key resistance factors in different tissues and plants (Yu et al., 2014; Huang et al., 2019).

### Glucosinolates

The involvement of TFs in modulating glucosinolate concentrations under heat stress has also been reported. Glucosinolates are secondary metabolites found almost exclusively in the Brassicaceae (Fahey et al., 2001). Although they are known to be present in all parts of the plant, their concentration varies with different tissues and developmental stages (Fieldsend and Milford, 1994). While seeds are considered the major compartment for their storage, their synthesis takes place after flowering in the leaves and the reproductive tissues such as the silique wall (Nour-Eldin et al., 2012). Some glucosinolates play protective roles in the plant stress defense mechanisms against biotic and abiotic stress (Ludwig-Müller and Krishna, 2000; Martínez-Ballesta et al., 2013). Elevated temperature was found to increase glucosinolate concentrations in *B. rapa*, with a noticeable involvement of BrMYB28 and BrMYB34 transcription factors in this process (Justen and Fritz, 2013). In *A. thaliana*, mutants deficient in glucosinolate metabolism experienced reduced expression of cytoplasmic Hsp90 and less thermostability under elevated temperature (Ludwig-Müller and Krishna, 2000), whilst the supply of two exogenous glucosinolate hydrolysis products increased the expression of HSPs and improved thermotolerance (Hara et al., 2013). Conversely, after heat treatment of 20d-old siliques of *B. napus*, Yu et al. (2014) reported a dramatic decrease in the expression of several transcripts involved in glucosinolate metabolism such as those involved in the indolic and benzenic pathways, suggesting the suppression of glucosinolate biosynthesis in the silique wall due to heat stress. This shows that down-regulation of such pathways under stressful conditions could act as a protective measure to conserve energy (Yu et al., 2014), but also to optimize redistribution of glucosinolates (Martínez-Ballesta et al., 2013), ensuring survival under adverse environmental conditions. Such opposite trends in glucosinolate concentrations in response to heat treatment and the involvement of distinct signaling molecules, such as TFs, reveal a complex stress response powered by various factors such as the intensity and duration of stress as well as the plant developmental stage. As little is known about the biological functions of glucosinolate metabolism under heat stress, there is a need for more research to further understand the auxiliary roles of individual compounds in modulating the plant physiological status. Thus, revealing the interconnectivity between different components and pathways involved in an effective heat tolerance system in *B. napus.*

## Impact of Polyploidy and Alternative Splicing on Heat Stress

Polyploidy or whole-genome duplication (WGD) is one of the major forces in plant evolution (Soltis et al., 2014). The resulting speciation and biodiversity have influenced the structure and evolution of plant genomics, giving rise to highly dynamic genomes with broad gene expression changes (Wang R. et al., 2019). Most of the world crops have undergone polyploidy including wheat, cotton, tobacco, oats, potato and coffee. A recent allopolyploidy event took place around 7,500 years ago between ancestors of *B. oleracea* and *B. rapa* resulting in the formation of the highly adaptive species *B. napus*. This in addition to earlier polyploidization events, has caused an aggregate of 72x genome multiplication since the origin of angiosperms (Chalhoub et al., 2014). These evolutionary events have led to the acquisition of strong and valuable agronomic traits, giving these species the ability to adapt and survive different climatic conditions. For example, one copy of *FLOWERING LOCUS C (FLC)*, a key adaptive gene controlling vernalization and photoperiod responses, exists in *A. thaliana*, while four copies exist in *B. rapa* and *B. oleracea* and nine or more in *B. napus* (Chalhoub et al., 2014).

There has been an increasing interest in studying the expression patterns of such duplicated genes. In polyploidy, duplicated genes may also be differentially spliced by the action of alternative splicing (AS) (Wang R. et al., 2019). This mechanism is characterized by the excision of introns and the production of differential exon combinations. The resulting genetic modifications alter transcript abundance and gene expression and eventually produce variant proteins (Liu Z. et al., 2018). During plant growth and development, AS plays key roles in different biological processes and most importantly, in response to biotic and abiotic stress (Guo et al., 2020). It is becoming increasingly evident that AS is not a by-product of stress but a regulatory part of the larger stress response (Kannan et al., 2018). Heat stress was found to induce an increase in AS events in genes that are associated with abiotic stress response such as those relating to protein folding (Chang et al., 2014; Kannan et al., 2018). In a recent study, analysis of differential AS landscape among A, B, and D wheat subgenomes under salt treatment found that homologous genes displayed differential AS responses under salt stress and more AS events were identified in the salt-sensitive cultivar than in the salt-tolerant one. Interestingly, specific AS events were found induced at different time points of salt stress (Guo et al., 2020). When multiple stresses were assessed, combined heat and drought stresses (HD) induced specific AS events compared with those induced by individual stresses. Upon comparison of differentially spliced genes (DSGs) and differentially expressed genes (DEGs), 40% of DSGs were overlapping with DEGs under heat stress and HD conditions (Liu Z. et al., 2018). These findings demonstrate the coordination between AS regulation and transcriptional regulation to orchestrate an effective stress response and ultimately contribute to abiotic stress tolerance. Similarly, in *B. napus*, RNA-Seq of plants subjected to cold, heat, and drought stress treatments also showed variation in expression and AS levels within the duplicated genes, with an overall A_T_ subgenome biases in gene expression and C_T_ subgenome biases in the extent of AS (Lee and Adams, 2020). Saminathan et al. (2015) hypothesized that polyploidy can influence AS, resulting in transcriptome modifications. In two independently synthesized lines of *B. napus*, alterations in AS events after polyploidy were reported (Zhou et al., 2011). Also, in response to heat, the number and type of AS events in the two related species *A. thaliana* and the highly thermotolerant polyploid *Boechera depauperate* varied significantly, suggesting a role for polyploidy in this variance. Moreover, this also demonstrates that AS responses to heat stress are species-specific (Kannan et al., 2018). In summary, the divergence in gene expression and AS patterns within the homologous genes may contribute to gene functional evolution which explains the flexibility of polyploids when responding to different abiotic stress (Zhang et al., 2010; Lee and Adams, 2020).

To further explore the role of AS in heat stress, Ling et al. (2018) investigated the existence of a “splicing memory” which is thought to be a key factor for achieving thermotolerance in Arabidopsis. As intron retention (IR) is the main AS form present in plants, the study aimed to investigate whether primed plants would preserve the same levels of these events during the recovery and memory-establishment phases and whether their response would differ from the response of non-primed plants after a recurrent exposure to heat. It was observed that heat-stressed plants accumulated unprocessed transcripts through splicing repression with marked levels of IR events, which eventually decreased to normal levels during recovery. In the second heat exposure, primed plants exhibited a very distinct response from non–primed plants. Primed plants remembered to undergo splicing and correctly processed transcripts similar to control plants under normal conditions. As a result, primed plants maintained a “splicing memory” that can carry out correct splicing and produce the necessary transcripts and proteins needed for the growth and development of plants after the cessation of stress, and subsequently ensuring the survivability of plants following another stress event (Ling et al., 2018). This further supports the role of AS an integral component in forming a priming-induced heat-stress memory in Arabidopsis (Sanyal et al., 2018). Further elucidation of this mechanism is needed to explore whether the discovered splicing-linked stress memory can be inherited across generations, or its function is limited somatically within an individual generation. Taken together, the polyploidy nature of *B. napus* and the regulatory role of AS in response to abiotic stress entail a further exploration of these evolutionary mechanisms under heat stress aiming to unveil novel species-specific interactions and to provide more understanding on its impact on thermotolerance, and more broadly, how polyploidy can impact the organismal response to heat stress.

## Dynamics of Epigenetic Modifications Under Heat Stress

In plants, high throughput technologies such as whole-genome bisulfite sequencing (WGBS) and chromatin immunoprecipitation (ChIP), and more recently nanopore technology, provide the opportunity to study epigenetic modifications in species and genotypes at genome-wide levels (Laird, 2010). Different types of epigenetic mechanisms exist and the most common are DNA methylation, histone modifications and small RNAs. Along with its vital role in maintaining genome integrity, increasing evidence indicates that epigenetic mechanisms in plants play a substantial part in modulating gene expression under different environmental conditions, including salinity, drought, extreme temperatures and exposure to heavy metals (Gao et al., 2014; Fan et al., 2020; Ueda and Seki, 2020).

### DNA Methylation

In higher plants, 30-50% of all cytosine residues of the nuclear DNA are methylated (Gao et al., 2014), and DNA methylation occurs in all three sequence contexts: the symmetric CG and CHG, and the asymmetric CHH, where H stands for A, C, or T (Li et al., 2016; Klungland and Robertson, 2017). Despite the availability of epigenetic research in *A. thaliana*, these studies are still very limited in Brassica crops. Different methylation levels can produce different profiles of gene expression and transcription, giving rise to different phenotypic responses. In plants, genome methylation status is maintained by the dynamic function of DNA methyltransferase and demethylase enzymes. These enzymes are classified into different categories depending on their protein structure and function (Liu G. et al., 2018). Fan et al. (2020) identified 22 methylase genes and six demethylase genes through studying two varieties of *B. napus* (YN171 and 93275; Table 1). In addition to the variations in methylation patterns detected at different sequence locations (gene regions and up/downstream regions) and contexts (CHG, CHH, and CG), analysis of gene expression in 22 tissues across different developmental stages showed that these genes have different regulatory roles in plant growth and development.

Several reports have demonstrated that plant adaptations to different abiotic stresses are associated with differences in methylation levels (Marconi et al., 2013; Dhar et al., 2014; Liu et al., 2017). In *B. napus*, a large set of genes were associated with changes in cytosine methylation in response to heat treatment. More specifically, this variation was prominent at the genotype level where more DNA methylation occurred in the heat-sensitive cultivar and more DNA demethylation occurred in the heat-tolerant one (Gao et al., 2014). The expression level of DNA methyltransferase and demethylase enzymes changed significantly in response to salt and heat stress, indicating that methylation of some genes is essential for the response to abiotic stress in plants (Fan et al., 2020; Table 1). Similarly, position- and context-dependent methylation variations were also seen in *B. rapa* under heat stress. Analysis of differential methylation and differential gene expression found that low-expressed genes were enriched in genes with high methylation levels and vice versa, except for mCG methylation in exon regions. Interestingly, different sets of differentially methylated DEGs were involved at the early and late stages of heat treatment (Liu G. et al., 2018).

During the reproductive stages, more complex relationships between DNA methylation and gene expression have also been elucidated. During the two pollen pathways (gametophytic development and pollen embryogenesis) and in response to heat treatment, *in vitro*-cultured microspores of *B. napus* changed their gametophytic developmental pathway toward embryogenesis through an epigenetic reprogramming control. This was associated with a decrease in global DNA methylation and activation of cell proliferation, while DNA methylation increased with pollen and embryo differentiation in a cell-type-specific manner (Solís et al., 2012). On the contrary, Li et al. (2016) reported that short term heat shock treatment induced a decrease in global DNA methylation in cultured microspores of *B. napus*, suggesting a lack of trend in the changes of DNA methylation under heat stress in different species or cell types.

### Histone Modifications

Histone modifications, including methylation, demethylation, and acetylation, are recognized as ubiquitous epigenetic marks that play a vital role in regulating chromatin structure and modulating gene expression (Xu et al., 2017). Other posttranslational modifications (PTMs), such as phosphorylation and ubiquitination have also been identified in transcriptional regulation in Arabidopsis (Füßl et al., 2018). The presence or absence of methylation on the lysine and arginine residues in histones affects their interaction with reader proteins and results in chromatin conformational changes. Eventually, these changes lead to either transcriptional activation or repression (Teperino et al., 2010). In plants, two Arg methylation sites (H3R17 and H4R3) and five Lys methylation sites (H3K4, H3K9, H3K27, H3K36, and H4K20) have been identified (Liu et al., 2010). In Arabidopsis, histone methylation involves both repressive (H4R3me2, H3K9me2/3, and H3K27me3) and active modifications (H4R3me2, H3K4me3, and H3K36me2/3) (Wang et al., 2016). In contrast to methylation, histone acetylation marks neutralize basic charges in histones, thus weakening histone/DNA interactions and making the DNA more accessible for the transcriptional machinery (Allis and Jenuwein, 2016). Histone modifications involve a complex network of epigenetic modifiers that serve to recognize, add or remove chemical moieties on histone tails or core domains (Xu et al., 2017). In response to stress, the combined activity of writers, readers and erasers regulate the level and type of histone modifications. Because tissue-specific expression is essential for stress response, these modifiers need to be recruited to specific loci for their participation. Various elements, such as: transcription factors and long non-coding RNAs have been proposed to act as recruiters of these regulators into specific chromatin sites (Deng et al., 2018). However, their role is still poorly explored in *B. napus*.

Recent studies have highlighted the role of histone modifications and their modifiers in response to abiotic stress. Alam et al. (2019) reported 18 *B. rapa* PHD (Plant homeodomain) finger genes as highly responsive to drought and salt treatments. As “epigenome readers,” PHD finger proteins can bind specifically to several histone modifications and confer activation or repression of the corresponding gene. Interestingly, some of these genes were found to be induced by more than 18-fold and others displayed different expression patterns at different time points of the treatments (Alam et al., 2019). In another study, 15 paralogous pairs of histone methyltransferase and demethylase genes also showed high variation in their expression profile in response to heat and cold stress where dynamic variations also took place in specific tissues and treatments, suggesting a complex role of these genes as robust candidates in the stress tolerance mechanisms of *B. rapa* (Liu G. et al., 2019).

Adding to this complexity, epigenetic regulators were found to interact with other stress-responsive elements to promote tolerance (Liu C. et al., 2019). To explore this relationship, Liu and colleagues investigated the role of polycomb repressive complex 2 (PRC2) in regulating ABA stress response at a genome level (Liu C. et al., 2019). PRC2 is one of the major families of polycomb group (PcG) factors that functions to induce a state of chromatin inactivation. Its core enzymes are responsible for histone H3 lysine-27 tri-methylation (H3K27me3), which is one of the major epigenetic marks that causes gene silencing (Liu C. et al., 2019). In *A. thaliana* more than 20% of genes are covered by H3K27me3 in one given organ (Lafos et al., 2011) and around 60% of protein-coding genes are silenced by H3K27me3 in different cell types (You et al., 2017). Therefore, H3K27me3 is considered a hallmark of gene repression, but its exact mechanism of action is not fully understood. H3K27me3 was found to preferentially target ABA-induced senescence-associated genes (SAGs) (Liu C. et al., 2019). ABA-induced senescence allows for the recycling and redistributing of nutrients to new growing organs and seeds, thus boosting the plant’s fitness and tolerance against abiotic stress (Zhao et al., 2016). In their study, Liu and colleagues found that mutants of PRC2 enzymes were hypersensitive to ABA. Accordingly, SAGs were derepressed and appeared highly induced in comparison to the wild type. This indicates that PRC2-mediated H3K27me3 plays a key role in attenuating the ABA response through buffering the expression of ABA-induced genes. Hence, fine tuning of ABA-induced senescence to protect the plant from oversensitive response (Liu C. et al., 2019). The role of PRC2 in regulating stress responses through modification of histones were also previously explored in different abiotic conditions such as drought, cold and salt where H3K27me3 modified the promotors of major stress response genes (Kwon et al., 2009).

### Non-coding RNAs

Another component of the epigenetic machinery thought to play a role in the response to abiotic stress are non-coding RNAs, which are involved in the regulation of gene expression, along with other roles in different biological processes (Zhang Y. C. et al., 2014). The short (20-24 nucleotides) small RNAs (sRNAs) and the long (more than 200 nucleotides) non–coding RNAs (lncRNAs) have no or limited discernible protein-coding functionalities.

Small RNAs include micro-RNAs (miRNAs) which are the most thoroughly characterized, and small interfering RNAs (siRNAs) (Bartel, 2009). sRNAs are known to affect gene expression *via* two main mechanisms: transcriptional (TGS) and posttranscriptional gene silencing (PTGS). To date, several studies have identified both conserved and novel miRNAs from various model plants such as Arabidopsis and rice in response to different abiotic stress, yet there is limited knowledge about the small RNA population in *B. napus* where only a small number of miRNAs have been identified and their role in response to heat stress is largely unknown (Jian et al., 2016). In a recent study, miRNAs mediated thermotolerance through the action of miR160 which altered the expression of heat shock proteins and improved seed germination and seedling survival under heat stress in *A. thaliana* (Lin et al., 2018). Additionally, five conserved miRNAs families along with four novel ones were found responsive to heat stress and resulted in either positive or negative regulation of their putative genes in *B. rapa* (Yu et al., 2012). Using computational prediction methods, Xie et al. (2007) were the first to study miRNAs in *B. napus* where they detected five miRNAs in response to auxin, cadmium stress and phosphate starvation. However, this study was carried out prior to the availability of a reference sequence which explains the small number of miRNAs detected by their approach. Subsequently, a set of differentially expressed miRNAs in response to sulfate deficiency and cadmium stress have been identified (Huang et al., 2010). High-throughput sequencing revealed a diverse and complex set of core miRNAs in *B. napus* that were involved in seed imbibition response to salt and drought stresses, suggesting the role of miRNAs in response to stressful conditions during seed germination (Jian et al., 2016). In *B. napus*, the regulatory role of miRNAs has been explored and detected in response to different abiotic stresses, yet little or no information is available in response to heat stress. Hence, more studies are warranted on this aspect to gain insight about their role in thermotolerance.

Non–coding RNAs were found to function as precursors of small RNAs (Joshi et al., 2016). They are grouped according to their genomic positions and orientations as follows: long intergenic non-coding RNAs (lincRNAs), long non-coding natural antisense transcripts (lncNATs), long intronic non-coding RNAs, and sense lncRNAs. In plants, lncRNAs research is still in its infancy with only a few studies showing their role in plant development and adaptation to abiotic stress (Jha et al., 2020). Nonetheless, lncRNAs have been found highly expressed in a tissue-specific or stage-specific manner, with some having preferential expression in the reproductive stages as well (Zhang Y. C. et al., 2014). There is increasing evidence about the role of lncRNAs as “biological regulators” for various processes including developmental, biotic and abiotic stress in animals and plants (Chekanova, 2015). To explore the current understanding of the participatory role of lncRNAs in the tolerance response to different abiotic stress conditions in plants, Jha et al. (2020) reviewed several studies that identified lncRNAs under drought, heat, cold, nutrient deficiency and metal toxicity. As a result, a plethora of differentially expressed heat stress lncRNAs and their corresponding heat stress-responsive target genes were reported from different crops. Song et al. (2016) identified 9,687 novel lncRNAs in *B*. *rapa* in response to heat treatment using RNA-seq. Likewise, two up-regulated lncRNAs (TalnRNA27 and TalnRNA5), which are miRNA precursors, were up-regulated in wheat in response to heat stress (Xin et al., 2010). During pollen development and fertilization, a systematic analysis in *B. rapa* revealed that 47 *cis*-acting lncRNAs and 451 *trans*-acting lncRNAs were highly co-expressed with their target genes (Huang et al., 2018). Furthermore, a co-expression network of *B. rapa* depicted 210 DEGs, 4 miRNAs and 33 lncRNAs under heat stress, where the biological functions identified through GO enrichment analysis revealed terms such as “brassinosteroid mediated signaling pathway”, “response to stimulus” and “response to heat”, suggesting their contribution in the heat response through these signaling pathways (Wang A. et al., 2019). At the seedling stage, two genotypes of *B. napus* exhibiting different sensitivity to drought were exposed to drought stress and rehydration treatment. Genome-wide analysis showed that the up-and down-regulated mRNAs co-expressed with lncRNAs participated in different metabolic pathways and were involved in different regulatory mechanisms, which suggested the existence of divergent mechanisms to modulate the response to drought stress and re-watering treatment (Tan et al., 2020). These findings show that lncRNAs in plants could form an additional layer of complexity to other stress response mechanisms during abiotic stress events. In *B. napus*, only a few studies were found addressing the roles of lncRNAs in response to certain stress conditions such as pathogens (Joshi et al., 2016), cadmium (Feng et al., 2016) and drought stress (Tan et al., 2020), while to the best of our knowledge, no studies have investigated the role of lncRNAs in responses to heat stress in this species. There is a significant knowledge gap concerning the roles of different epigenetic mechanisms in response to heat stress in *B. napus*. Owing to its complexity and poorly understood mechanisms, a deeper knowledge of these intricate epigenetic components would serve as a stepping-stone to gain insights into the genomic regulation behind heat stress-mediated responses in this important crop species.

## Systems Biology Approaches in *Brassica napus* Under Heat Stress

Omics approaches are now routinely used in helping to understand plant responses and adaptations to different biotic and abiotic conditions. However, their exploitation in *B. napus* is limited and the knowledge of the impact of heat stress on its yield determining reproductive stages is still fragmentary. Nevertheless, the availability of reference genomes in Brassica species has contributed to major improvements in understanding the genetics of these crops. This has created a foundation for identifying possible interactions that link genotypes and phenotypes. Subsequently, emerging genomics and transcriptomics studies have resulted in the development of transgenic cultivars with potentially stronger agronomic properties than their non-transgenic cultivars (Raza et al., 2021). In *B. napus* and other related species, several genomic projects have been conducted to study the effect of different abiotic stresses on different genetic and physiological properties. Using GWAS, numerous studies examined genomic regions and quantitative trait loci (QTL) controlling various traits that are affected by heat stress (Bac-Molenaar et al., 2015; Rahaman et al., 2018). Others identified transcription factors that play regulatory roles in abiotic stress responses including heat (Yoshida et al., 2008; He et al., 2016; Wang et al., 2018; Liang et al., 2019; Ghorbani et al., 2020) and some looked at gene families that are involved in controlling different hormonal signaling networks during heat stress (Justen and Fritz, 2013; Di et al., 2018).

At the transcript level, global transcriptome profiling allows for dynamic quantification of gene expression and their isoforms at different developmental stages in different tissues (Wang et al., 2009). For instance, combined RNA-seq and qRT-PCR identified and analyzed the expression patterns of 64 genes encoding BnaHSF proteins in the *B. napus* genome across different tissues and under heat, drought and high CO_2_ stresses (Zhu et al., 2017; Table 1). Similarly, organ-specific expression was detected using transcription profiling in *B. napus* siliques at the seed-filling stage, where some genes were preferentially expressed in heat-stressed silique walls or seeds and others were involved in specific biological functions of each tissue, such as glucosinolate metabolism in silique wall and flavonoid synthesis in seeds (Yu et al., 2014).

Examining the impact of high night temperature on the fatty acid composition of *B. napus* seeds, in-depth RNA-seq analysis revealed up-regulated gibberellin signaling which was also associated with active expression of genes involved in fatty acid catabolism and glyoxylate metabolism pathways (Zhou et al., 2018). Furthermore, a genome-wide analysis allowed the identification and functional analysis of 321 putative AP2/ERF TFs belonging to five major subfamilies, including DREB, ERF, AP2, RAV, and BnSoloist in *B. napus* (Owji et al., 2017). Gene expression analysis using RNA-seq revealed a high expression of AP2/ERF genes in the roots as compared to other organs. Gene ontology annotation, regulatory elements identification, and functional association network showed that most of these TFs are responsive to different abiotic stresses such as drought, salinity, and cold and play significant roles in the developmental processes, particularly organ speciation and embryogenesis (Owji et al., 2017).

Plant responses to heat are depicted by many quantitative factors such as time, intensity and duration of stress. At the molecular level, these responses result in profound structural and metabolic modifications, ranging from disruption of membrane and cytoskeleton stability to metabolic alterations of enzyme activity and accumulation of toxic metabolites such as reactive oxygen species (ROS). Although genomic and transcriptomic studies using RNA-seq have offered revolutionary approaches to interpret genomic data, these data do not always reflect the exact proteomic and metabolic state as gene products are also influenced by other processes such as post-translational and co-translational modifications. Hence, integration of metabolic and proteomic approaches will further help to better understand the events taking place at the molecular level (Rocco et al., 2013). Recently, omics approaches have been fine-tuned to elucidate the abiotic stress-related genes and develop tolerant cultivars that are capable to withstand current climatic and environmental changes (Raza et al., 2021). For example, studying the proteome response of a crop species during high-temperature stress will help to reveal the proteins and their corresponding biological mechanisms responding to these stresses. Moreover, as proteins act as direct effectors of phenotype, this proximity allows the prediction of affected phenotypes and hence, improving the breeding outcomes (Kosová et al., 2015). Although the proteome comprises of all protein components in a living organism, this set of proteins varies considerably depending on the developmental stage, growth conditions, tissue and cell type, giving rise to infinite proteomes within one organism (Kosová et al., 2015). Therefore, to gain a comprehensive insight into the protein profiles and pathways that are associated with heat, extensive research needs to be carried out not just at the organismal level but also looking at specific developmental stages and growth conditions.

Although numerous proteomic studies addressing the impact of various abiotic stresses in model species and various crops have been reported and reviewed (Benešová et al., 2012; Hossain et al., 2012; Kosová et al., 2013; Li et al., 2013), there is very limited knowledge to date on the proteome of *B. napus* under heat stress. The existing studies have identified proteins that are thought to be involved in thermotolerance, redox homeostasis, carbohydrate metabolism and protein synthesis and degradation (Lee et al., 2007; Neilson et al., 2010). In *A. thaliana*, analysis of the protein repertoire in the rosette leaves after exposing seedlings to short-term heat stress revealed differentially represented proteins that grouped into two main broad classes including energy and metabolism and stress response proteins (Rocco et al., 2013). In the first proteomic report in *B. napus* in response to heat stress, Ismaili et al. (2015) observed an increase in the abundance of ascorbate peroxidase protein and its corresponding mRNA transcript, suggesting its role in short-term high-temperature stress response. Also, the up regulation of proteins involved in energy and metabolism is thought to have utilized most of the nutritive reserves in the seedlings, resulting in a noticeable reduction in growth as a response to heat (Ismaili et al., 2015). Similarly, the first proteome analysis of *B. napus* response to drought stress also reported a reduction in growth that was associated with decreased abundance of HSP 70 and tubulin beta-2 proteins in the drought-sensitive and hybrid F1 lines (Mohammadi et al., 2012). Using yeast two-hybrid assay, the interactions among various Calcineurin B-like proteins (CBLs) and CBL-interacting protein kinases (CIPKs) were demonstrated in *B. napus* under several abiotic stresses (Zhang H. et al., 2014). Furthermore, expression levels of six selected *BnaCBLs* and 12 *BnaCIPKs* genes in response to salt, drought, cold, heat, and ABA showed that the two gene families were responsive to multiple stimuli, suggesting that the CBL-CIPK network in *B. napus* may be a common link for a variety of signaling pathways (Zhang H. et al., 2014). Yan et al. (2016) used a comparative proteomic approach to screen for thermotolerance genes in seeds of *B. napus*. As a result, a *B. napus* GLYI gene was identified. GLYI is a member of glyoxalase system that plays a role in methylglyoxal detoxification during carbohydrate and lipid metabolism. Two-dimensional gel analyses showed that the abundance of BnGLYI-3 protein in thermotolerant seeds significantly increased in response to heat stress, suggesting its role in inducing thermotolerance to high temperature stress in *B. napus* seeds (Yan et al., 2016).

Along with proteomics and other omics approaches, metabolomics has emerged as an exciting scientific discipline where cellular metabolites are profiled to reveal connections among genes, phenotypes and other cellular biomarkers in different biological species (Raza, 2020). The metabolome is the total pool of metabolites present in an organism (Razzaq et al., 2019). Through metabolomics studies, it is now possible to identify and measure primary and secondary metabolites to characterize genetic or environmental variations in plants (Razzaq et al., 2019). Under heat stress conditions, metabolomics provides a systems biology approach to understand complex metabolic networks and enhance thermotolerance and can be a useful tool in distinguishing between genotypes with different heat sensitivity and understanding how changes in the transcriptome and proteome manifest themselves at the cellular and tissue levels. Koscielny et al. (2018) identified 25 metabolic markers that discriminated between the heat-tolerant and -susceptible genotypes of *B. napus* during the reproduction stage under heat stress, through an untargeted metabolic assessment using gas chromatography-mass spectrometry. The latter finding demonstrates how genetic variations exhibited by different genotypes are translated into distinct agronomic traits that eventually allow its survivability under suboptimal conditions. In addition, metabolome information collected from stressed plants has also helped define the severity and timing of heat stress (Koscielny et al., 2018). In another metabolomic study, numerous unknown potential regulatory relationships involved in glucosinolate metabolism were identified through a doubled-haploid mapping population of *B. napus*. Feng et al. (2012) demonstrated the impact of genetic variation on the biochemical pathways through combining genetic and metabolic analysis of different glucosinolate components in the leaves and seeds. Subsequently, this has resulted in the identification of 105 metabolite QTLs and the construction of an advanced metabolic network for the glucosinolate composition in both leaves and seeds (Feng et al., 2012). Integration of metabolomic approaches with other omics tools has therefore the potential to reveal crucial interactions and regulatory networks between genes, proteins, and many essential regulatory components, where different responses meet to form a complex machinery of plant stress tolerance.

## Conclusion

Despite current advances in exploring the impact of different biotic and abiotic stress factors within different Brassica species, this review has identified several knowledge gaps regarding the impact of heat stress on *B. napus* during its yield determining reproductive stages, where in-depth analysis of this subject is still needed. The opposite trends in expression of key genetic and epigenetic components identified in different species and in cultivars within the same species under various abiotic stresses call for further research in this field. Moreover, variations in intensity, time and duration of heat stress have been found to generate modified responses in plants. Therefore, to understand its impact on *B. napus* crops in specific geographical areas, regional studies on local cultivars should take place to simulate field conditions specific to these areas. To date, only a few studies have addressed the impact of heat stress on the metabolome and proteome of *B. napus*, and the underline cellular mechanisms are still poorly understood. On this account, combining genetic studies with different omics approaches will provide the means to explore the intricate responses arising following exposure to heat stress and accomplish a system-level understanding of how heat stress alters the yield and quality of the *B. napus* crop. It is expected that the findings of these studies will help breeders to produce cultivars with strong thermotolerance traits and to mitigate the detrimental effect of global warming on agriculture through contributing to sustainable food production and security.

## Author Contributions

FM, FR, MA, and JH conceived the research. MK undertook the review of the literature, prepared the figures, and drafted the manuscript. All authors contributed to and revised the manuscript, given the opportunity to comment, and approved the final version of the manuscript.

## Conflict of Interest

The authors declare that the research was conducted in the absence of any commercial or financial relationships that could be construed as a potential conflict of interest.

## Publisher’s Note

All claims expressed in this article are solely those of the authors and do not necessarily represent those of their affiliated organizations, or those of the publisher, the editors and the reviewers. Any product that may be evaluated in this article, or claim that may be made by its manufacturer, is not guaranteed or endorsed by the publisher.
